# Off-label prescribing of quetiapine in south locality crisis teams

**DOI:** 10.1192/bjo.2021.267

**Published:** 2021-06-18

**Authors:** Mamta Kumari, Arun Kumar Gupta, Peter Clarke

**Affiliations:** 1 Roseberry Park Hospital; 2 Sunderland South CTT; 3 South Pharmacy Department, Hopewood Park

## Abstract

**Aims:**

The audit was carried out to determine the frequency of off label prescribing of quetiapine and compliance with standards within Trust Policy (UHM PGN 02 PPT PGN 08) – Physical Health Monitoring of Patients Prescribed Antipsychotics and other Psychotropic Medicines, NICE CG178, General Medical Council Ethical Standards and Royal College of Psychiatrists – College Report CR210.

The main objectives of the audit were to determine if:

Patients have been appropriately informed of off-label status and consent recorded.

Alternative licensed treatment first used/ruled out.

Appropriate communication on transfer of care.

Appropriate physical health monitoring completed.

**Background:**

Quetiapine is associated with various physical side effects. Patients should be fully informed of the expected risks and benefits of treatment, and the limited evidence base for off-label prescribing.

There are additional issues around the transfer of prescribing to primary care.

**Method:**

The sample consisted of 50 consecutive patients selected from the crisis team caseload in the month of December 2018.

Data reviewed in this audit were taken from six months period.

Records audited were obtained from RiO (electronic records) and prescription charts.

Data collection was started in January 2019 and completed in March 2019

The audit tool was a dichotomous scale questionnaire based on NICE guidelines.

**Result:**

4 patients from the sample (8%) were prescribed off-label quetiapine.

100% had physical health monitoring completed as per Trust policy.

100% off-label indication been clearly documented in notes.

100% Consent to treatment was documented.

100% had medication reviewed in the previous 6 months.

75% had licensed medication used or ruled out before considering off-label quetiapine use

25% risks/benefits of treatment were documented as part of a patient discussion.

25% had documented evidence that alternative treatment options were discussed.

25% had documented evidence of Community consultant/GP consent/agreement was obtained before transfer of prescribing

75% had a documented plan for review of quetiapine for treatment efficacy and side effects

50% had a documented plan in place for ongoing physical health monitoring

**Conclusion:**

Suggested a wider audit may be required with greater patient numbers and which specifically filters for patients prescribed quetiapine.

Audit result has been shared with Crisis team members, Medicines Optimisation Committee and South Locality Quality Standards Committee in the trust.

